# Ten Year Review of Pulmonary Nocardiosis: A Series of 55 Cases

**DOI:** 10.7759/cureus.4759

**Published:** 2019-05-26

**Authors:** Khurram Zia, Taha Nafees, Muhammad Faizan, Osama Salam, Syeda Ifra Asad, Yasir A Khan, Ahmed Altaf

**Affiliations:** 1 Internal Medicine, Liaquat College of Medicine and Dentistry, Karachi, PAK; 2 Internal Medicine, Karachi Medical and Dental College, Karachi, PAK; 3 Internal Medicine, Dow University of Health Sciences, Karachi, PAK; 4 Miscellaneous, Dow University of Health Sciences, Karachi, PAK; 5 Internal Medicine, Dr. Ziauddin Hospital, Karachi, PAK

**Keywords:** pulmonary nocardia, nocardiosis, hiv, rare infection, case series, pulmonary infections, pakistan, chronic steroid therapy

## Abstract

Introduction

Nocardiosis is a rare opportunistic bacterial infection usually seen in immunosuppressed patients. It is caused by gram-positive, aerobic actinomycetes of the Nocardia genus. The most common site of infection is lungs; but it may affect other organs or even disseminate into blood.

Methods

In this a 10-year retrospective review, all diagnosed cases of Pulmonary Nocardiosis in a tertiary care hospital were included. The clinical and radiological characteristics, course of complications and lifesaving interventions, and disease outcome were evaluated.

Results

Among the 55 identified cases, most common risk factor was chronic steroid therapy (n=38; 69.1%). Among respiratory diseases, chronic obstructive pulmonary disease (n=13; 23.6%) and tuberculosis (n=12; 21.8%) were the most common. On chest radiograph, pleural effusion (n=23; 41.8%) and consolidation (n=22; 40.0%) were the common findings. Complications were observed in 32 (58.2%) patients with septicemia and respiratory failure being the most common (n=15; 46.8% in each). Dissemination occurred in 10 (31.2%) patients. The mortality rate of Nocardia is 34.5% (n=19).

Conclusion

The disease burden of Nocardia is underestimated by clinicians and researchers. Pulmonary Nocardia should always be a differential diagnosis of signs of lower respiratory tract infection and must be excluded in patients not responding to treatment of chronic obstructive pulmonary disease (COPD) and pulmonary tuberculosis. Early recognition and individualized management plan can ensure successful recovery.

## Introduction

Nocardia species belong to a diverse group of bacteria known as aerobic actinomycetes. The genus Nocardia are gram-positive organisms that are partially acid-fast due to the mycolic acid content of their cell wall. Nocardia is common in the environment worldwide, including various soil, freshwater, marine-water, and organic-matter habitats, where they are believed to maintain a saprophytic existence. Nocardia species may also be present in domestic environments such as house dust, garden soil, beach sand, and swimming pools [1]. Airborne droplets are the most common method of contracting the infection, making lungs the most commonly affected organs. Pulmonary nocardiosis forms up to 70% of all cases of nocardiosis [2].

In most instances, Nocardia is an opportunistic pathogen, with the majority of diagnosis made in immunocompromised hosts, including those with long-term corticosteroid exposure, malignancy, human immunodeficiency virus (HIV) infection, and history of transplantation [1]. The incidence of nocardiosis is over 100% in immunocompromised individuals as compared to the normal population [2]. Globally, the data regarding the incidence of Nocardia is limited. There hasn’t been any large scale study to estimate the global prevalence or incidence of the infection. However, numerous case reports and case series have been reported [3-4]. Prevalence of Nocardia in Pakistan is not known and the publications regarding pulmonary nocardiosis in the medical literature are also limited to just case reports, with few case series, which also includes cases of extra-pulmonary nocardiosis [5-6].

With the rise in cases of chronic respiratory infections, malignancies, and transplantations, opportunistic pathogens in these high risk groups are becoming a major health concern for the clinicians [7]. The aim of this study is to highlight the clinical experience of a major tertiary care hospital in diagnosing and managing pulmonary nocardiosis.

## Materials and methods

It was a retrospective analysis of all diagnosed cases pulmonary nocardiosis recruited from the archives of Pulmonology department of a tertiary care hospital in Karachi. Records included in this study were from January 1, 2001 through December 31, 2010. The study was approved by the institutional review committee.

There were 55 identified cases of Nocardia. All cases were diagnosed on sputum gram staining and culture. Patient age, gender, co-morbidity, presenting complains, chest x-ray findings, and course of clinical outcome was noted from their medical records. Medical complications (if occurred) and urgent life-saving interventions (if taken) were also included. Rate of mortality was also calculated. Data were processed through SPSS version 22.0 (NY: USA). Frequencies and percentages were calculated for all categorical characteristics.

## Results

Among the 55 identified cases, 42 (76.4 %) were males and 13 (23.6%) were females. Mean age of the cases was 49.18 ± 9.45 years.

The most common predisposing risk factor was chronic steroid therapy (n=38; 69.1%). Among respiratory diseases, chronic obstructive pulmonary disease (COPD) (n=13; 23.6%) and tuberculosis (n=12; 21.8%) were the most common. Human immunodeficiency virus (HIV) infection was present in five (9.1%) cases. Other risk factors along with the presenting complains are shown in Table 1.

**Table 1:** Predisposing risk factors and presenting complaints seen in patients diagnosed with Nocardia (n=55) *Patients may have more than one risk factor COPD, Chronic obstructive pulmonary disease; HIV, Human immunodeficiency virus

Predisposing risk factor*	Frequency (%)	Presenting complains*	Frequency (%)
Chronic steroid therapy	38 (69.1%)	Cough	51 (92.7%)
Malignancy	15 (27.3%)	Fever	44 (80.0%)
COPD	13 (23.6%)	Dyspnea	35 (63.6%)
Tuberculosis	12 (21.8%)	Crepitation	30 (54.5%)
Organ transplant	10 (18.2%)	Wheeze	19 (34.5%)
HIV infection	5 (9.1%)	Hemoptysis	18 (32.7%)
Chronic Asthma	4 (7.3%)	Weight loss	18 (32.7%)
Bronchiectasis	4 (7.3%)	Chest pain	16 (29.1%)
Alcoholism	2 (3.6%)	Decreased Appetite	14 (25.5%)
		Hepatomegaly	2 (3.6%)

Unfortunately, none of the isolates were sub-speciated beyond Nocardia spp. There was no sensitivity data reported on any of the species isolated. Sputum was the most frequent site for the isolation of Nocardia spp. (n=49; 89.1%). For cases with non-productive cough; sputum was induced by hypertonic saline nebulization (n=11; 20%); if that also failed, bronchoalveolar lavage (BAL) was obtained via bronchoscopy (n=3; 5.4%). The pleural fluid tap was obtained in 6 (10.9%) patients.

Chest X-ray was done periodically over the course of the hospital stay. On first chest X-ray, at the time of admission, pleural effusion was seen in 23 (41.8%) patients, consolidation was seen in 22 (40.0%) patients, localized infiltrates were seen in 19 (34.5%), diffused infiltrates were seen in 14 (25.5%), and pneumothorax was present in seven (12.7%) patients.

Complications were observed in 32 (58.2%) patients. Most common being septicemia and respiratory failure in 15 (46.8%) patients each. Dissemination occurred in 10 (31.2%) patients. Dissemination to the skin was found in one patient, to the central nervous system in three patients, and to the blood in six patients (Table 2).

**Table 2:** Disease-related complications during hospital stay in diagnosed cases of pulmonary Nocardia (n=32) *Patients may have developed more than one complications

Complications*	Frequency (%)
Respiratory failure	15 (46.8%)
Septicemia	15 (46.8%)
Empyema	11 (34.3%)
Dissemination	10 (31.2%)
Pneumothorax	4 (12.5%)
Subcutaneous emphysema	3 (9.3%)

There were 35 patients who were critical and needed lifesaving interventions. The most frequent of these was endotracheal tube placement for mechanical ventilation secondary to respiratory failure in 15 (42.8%) patients followed by chest tube placement in 13 (37.1%) patients and decortication in six (17.1%) patients (Table 3).

**Table 3:** Lifesaving interventions taken in patients with Nocardia (n=35) *One or more lifesaving interventions may have been taken in one patient ETT, endotracheal tube; EET, endoesophageal Tube; VATS, video-assisted thoracoscopic surgery

Lifesaving interventions*	Frequency (%)
ETT placement	15 (42.8%)
Emergency chest tube placement	13 (37.1%)
Decortication of lung via VATS	6 (17.1%)
EET with tracheostomy	5 (14.2%)

There were 36 (65.4%) patients who were cured and there were 19 deaths making the mortality rate of Nocardia 34.5%. The common causes of mortality included cardiopulmonary arrest secondary to septicemia or respiratory failure (n=11; 57.8%), failure to extubate (n=6; 31.5%), and pulmonary embolism (n=2; 10.5%). In patients with dissemination, mortality was 100%.

## Discussion

This retrospective analysis provides a detailed clinical picture of Pulmonary nocardiosis in Pakistan. Chronic steroid therapy, malignancy, and respiratory infections including tuberculosis are common predisposing conditions. Respiratory failure and the need for mechanical ventilation is high. Dissemination is common. The mortality rate is high.

The major shortcoming in this study is its retrospective methodology. It is not only subjected to recall bias but also the medical records were not as detailed. Histopathology department couldn’t be taken on board; hence, the frequency of Nocardia species couldn’t be assessed in this analysis. The study reports statistics from pulmonary department only, hence, extra-pulmonary Nocardia cases are missing.

Immunosuppression due to chronic steroid use was the most common predisposing risk factor for Nocardia in this study. Among respiratory tract diseases, COPD was the most common. Studies have reported that COPD is the disease most often treated with corticosteroid therapy and therefore leaves the patient prone to Nocardiosis infection more than any other respiratory condition; even without steroid therapy COPD remains a critical risk factor behind nocardiosis [8-9]. Munoz et al. in their series of 27 Nocardia cases identified 70% of their patients to have COPD [10]. In a 13-year long analysis, the most common condition associated with pulmonary nocardiosis was treatment with steroids (64.5%), followed by organ transplantation (29%), COPD (23%), HIV infection (19%), and alcohol intake (6.5%) [11].

The crucial problem with Nocardia is the variety of symptoms it can present. The symptoms are nonspecific and follow a chronic course [12]. Even at the time of presentation, symptoms have been there for days or even years. Even on chest radiographs, nonspecific and pleomorphic manifestations are seen. Chest radiographs may range from consolidations and cavitary nodules to masses and interstitial patterns with upper lobes more frequently affected [13]. Lack of any cardinal clinical or radiological sign, difficult microbiological diagnosis, and its rarity take Nocardia down on the list of differential diagnoses of a clinician. Missed diagnosis leads to delay in therapy which is consequently a predictor of higher mortality in these patients [13-14].

Due to misdiagnosis and high mortality, there remains a paucity of clinical trials on pulmonary Nocardia. There are also no standard treatment guidelines for managing this infection. Keeping in view the difference in virulence and drug susceptibility, treatment strategies are customized. The most common first-line therapy for Nocardia is trimethoprim-sulfamethoxazole at 25-50 mg/kg per day in divided doses. Based on drug susceptibility other antibiotics include amikacin, ceftriaxone, levofloxacin, linezolid, imipenem, and amoxicillin-clavulanic acid. The recommended treatment duration is six to twelve months; however, may be prolonged if the patient is immunocompromised [1].

The burden of pulmonary nocardiosis seems to be highly underestimated. The disease should always be considered in the differential diagnosis of pneumonia, not only in the immunocompromised but also in the immunocompetent, especially when they are not responding to the standard therapy. In tuberculosis-endemic countries like India, nocardiosis should always be excluded among patients not responding to anti-tubercular treatment. Early recognition and appropriate individualized treatment is the key to a successful. 

## Conclusions

It is likely that the researchers and clinicians are underestimating the prevailing burden of Pulmonary Nocardiosis. It is no longer a disease-specific for immunocompromised patients, but is also appearing among the immunocompetent ones. Pulmonary Nocardia should always be a differential diagnosis of signs of lower respiratory tract infection. In countries with a high prevalence of lower respiratory tract diseases including COPD and pulmonary tuberculosis, Nocardia should always be excluded in non-responsive patients. Although the disease is associated with a high mortality rate, early recognition and individualized management plan can ensure successful recovery in these patients.
